# Supplementary material for: Combined Menin and XPO1 inhibition drive synergistic antileukemic activity in KMT2Ar and NPM1-m AML
Source: bioRxiv. 2026 Mar 13:2026.03.10.710924. Preprint. [Version 1] doi: 10.64898/2026.03.10.710924 (PMC13060913; doi:10.64898/2026.03.10.710924)
Supplement: Supplement 1 [file media-1.pdf]

Figure S1

Interactions

Gene ID A	Gene name A	Gene ID B	Gene name B	Organism	Type	Source	Score ?
ENSG00000133895	MEN1	ENSG00000283094	PTEN	H. sapiens	SSL	Slorth	0.886437
ENSG00000100387	RBX1	ENSG00000133895	MEN1	H. sapiens	SSL	Slorth	0.886197
ENSG00000049759	NEDD4L	ENSG00000133895	MEN1	H. sapiens	SSL	Slorth	0.885932
ENSG00000082898	XPO1	ENSG00000133895	MEN1	H. sapiens	SSL	Slorth	0.885585
ENSG00000133895	MEN1	ENSG00000165392	WRN	H. sapiens	SSL	Slorth	0.885379

Figure S2

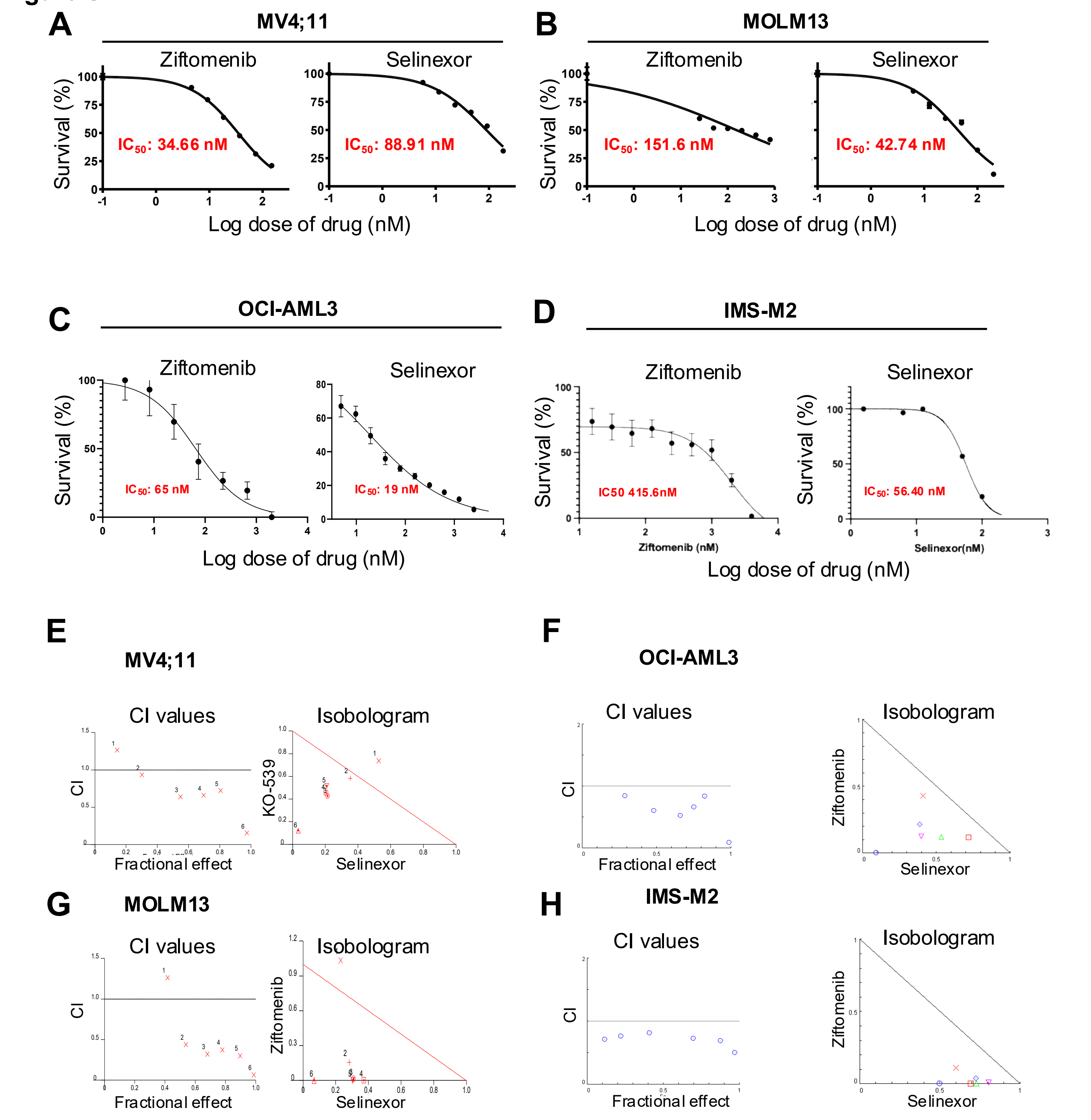

I

Combination Index (CI)-IMS-M2

Ziftomenib (nM)	Selinexor (nM)	Effect	CI
4000.0	160.0	0.97099	0.50124
2000.0	80.0	0.87764	0.69317
1000.0	40.0	0.69872	0.73145

J

Combination Index (CI)-OCI-AML3

Ziftomenib (nM)	Selinexor (nM)	Effect	CI
2000	160	0.82549	0.83803
1000	80	0.75247	0.65992
500	40	0.66361	0.52290

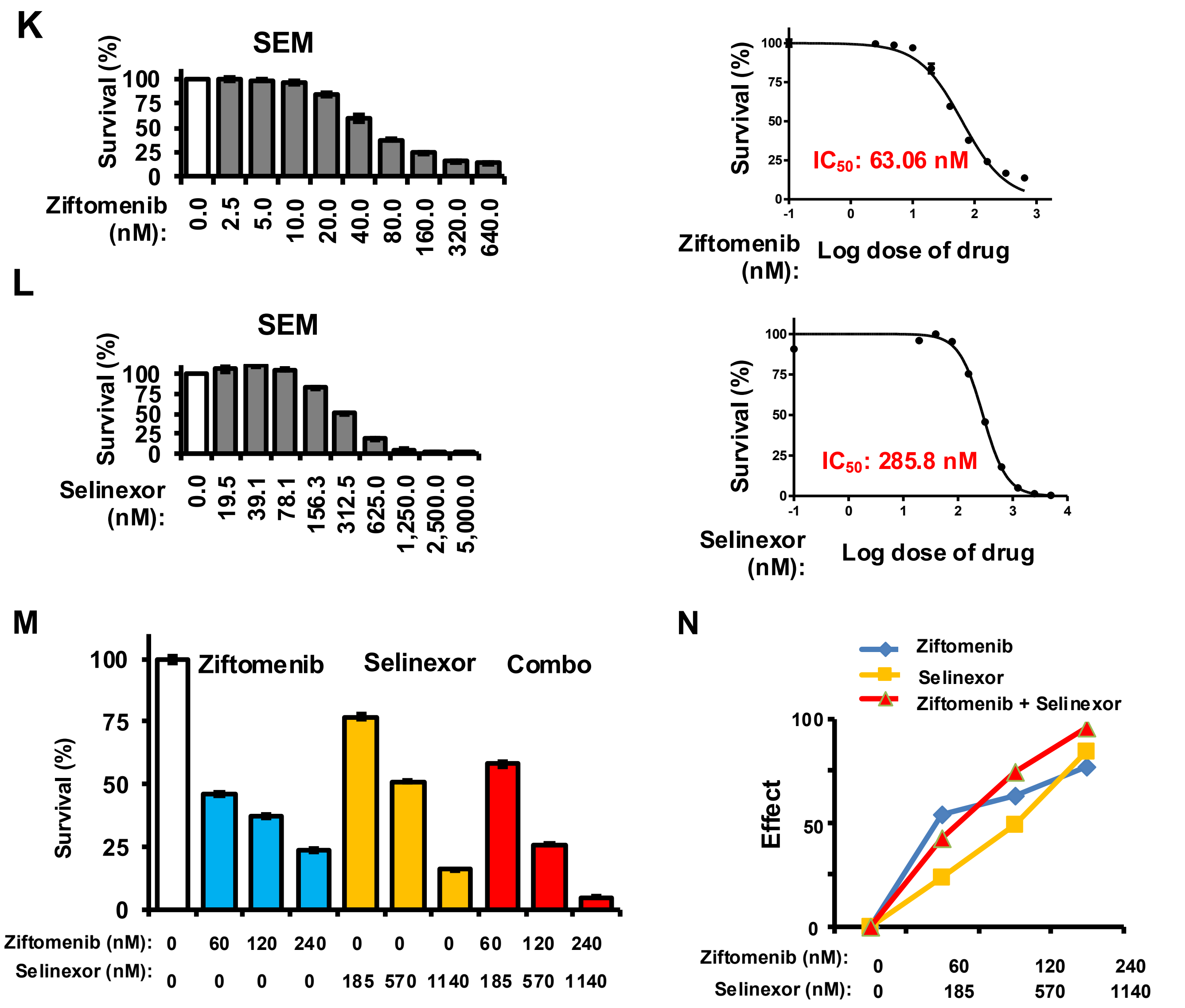

O

Combination Index (CI)-SEM

Ziftomenib (nM)	Selinexor (nM)	Effect	CI
60	285	0.419234	2.429
120	570	0.74289	1.194
240	1140	0.952881	0.580

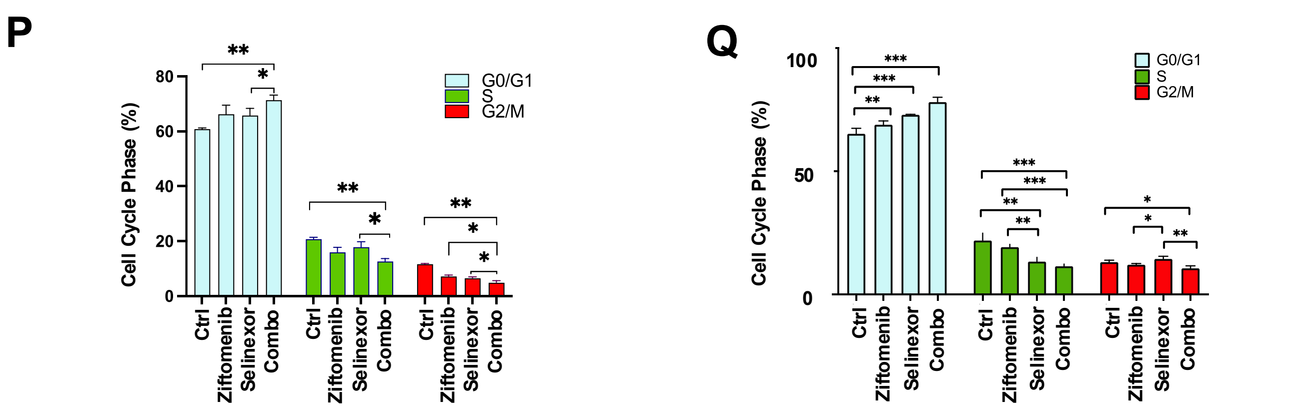

Figure S3

A

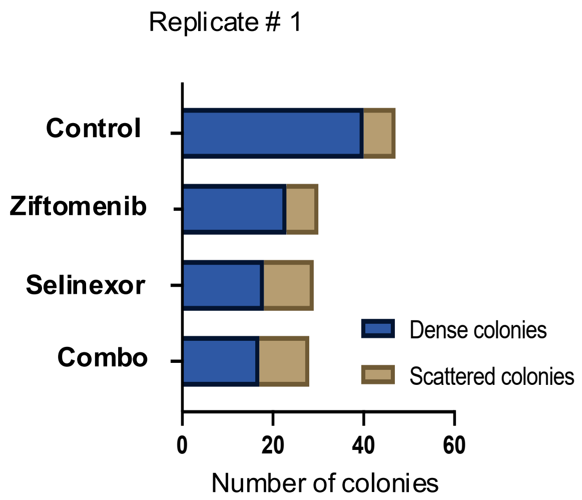
Replicate # 1
Control
Ziftomenib
Selinexor
Combo
Dense colonies
Scattered colonies
0
20
40
60
Number of colonies

B

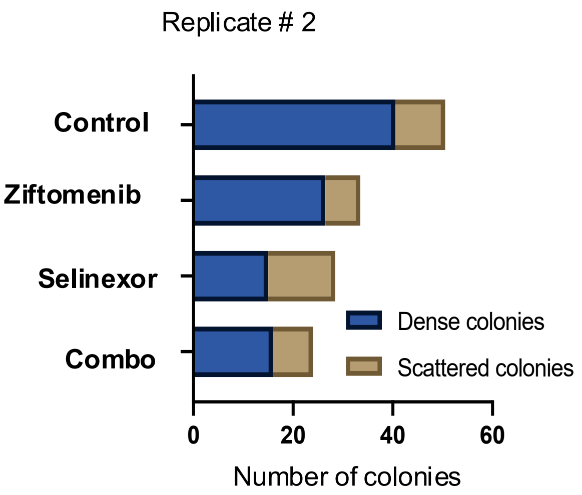
Replicate # 2
Control
Ziftomenib
Selinexor
Combo
Dense colonies
Scattered colonies
0
20
40
60
Number of colonies

Figure S4

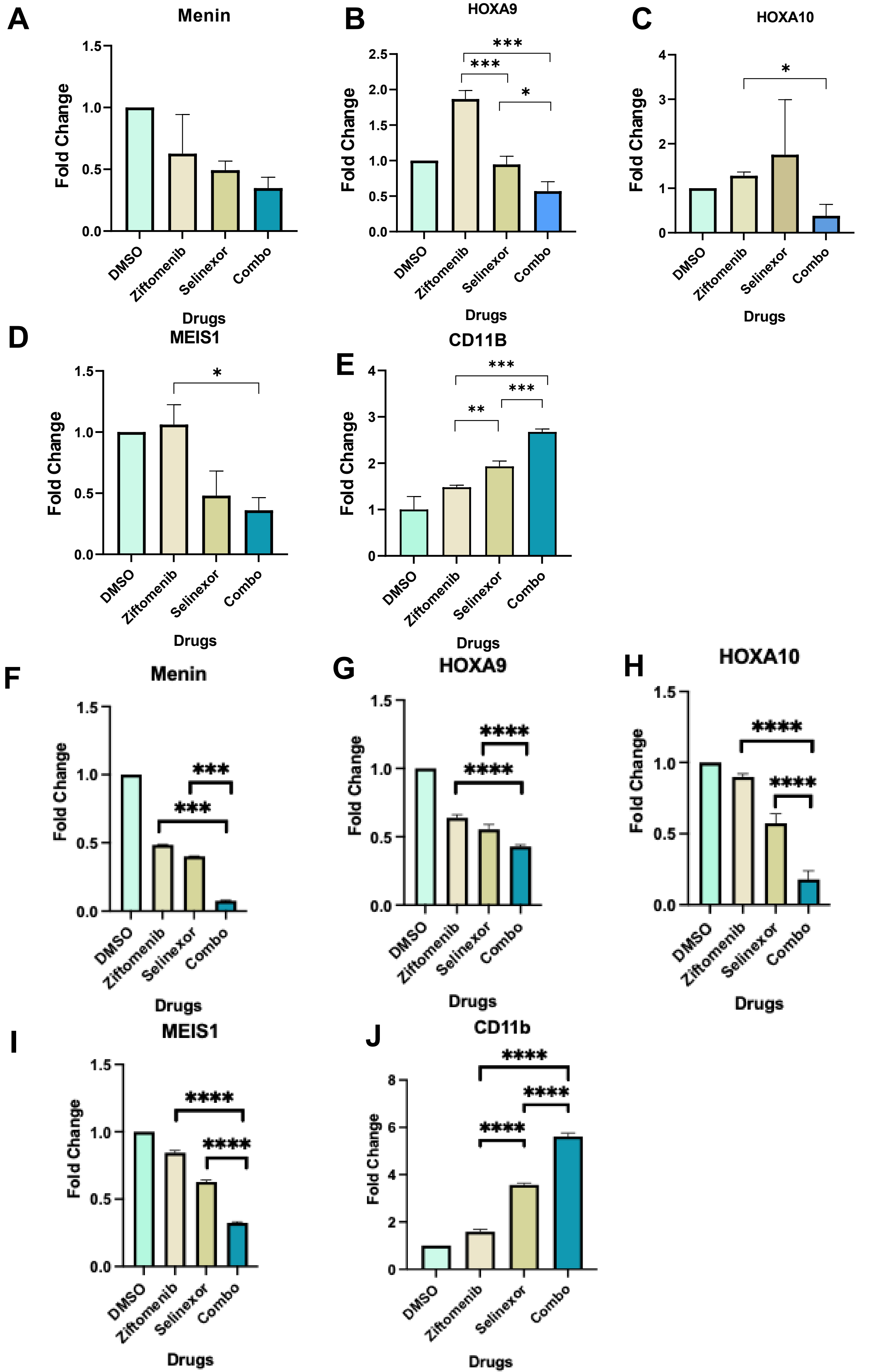

A
Menin
Fold Change
1.5
1.0
0.5
0.0
DMSO
Ziftomenib
Selinexor
Combo
Drugs
B
HOXA9
Fold Change
2.5
2.0
1.5
1.0
0.5
0.0
***
***
*
DMSO
Ziftomenib
Selinexor
Combo
Drugs
C
HOXA10
Fold Change
4
3
2
1
0
*
DMSO
Ziftomenib
Selinexor
Combo
Drugs
D
MEIS1
Fold Change
1.5
1.0
0.5
0.0
*
DMSO
Ziftomenib
Selinexor
Combo
Drugs
E
CD11B
Fold Change
4
3
2
1
0
***
***
**
DMSO
Ziftomenib
Selinexor
Combo
Drugs
F
Menin
Fold Change
1.5
1.0
0.5
0.0
***
***
DMSO
Ziftomenib
Selinexor
Combo
Drugs
G
HOXA9
Fold Change
1.5
1.0
0.5
0.0
****
****
DMSO
Ziftomenib
Selinexor
Combo
Drugs
H
HOXA10
Fold Change
1.5
1.0
0.5
0.0
****
****
DMSO
Ziftomenib
Selinexor
Combo
Drugs
I
MEIS1
Fold Change
1.5
1.0
0.5
0.0
****
****
DMSO
Ziftomenib
Selinexor
Combo
Drugs
J
CD11b
Fold Change
8
6
4
2
0
****
****
****
DMSO
Ziftomenib
Selinexor
Combo
Drugs

Figure S5

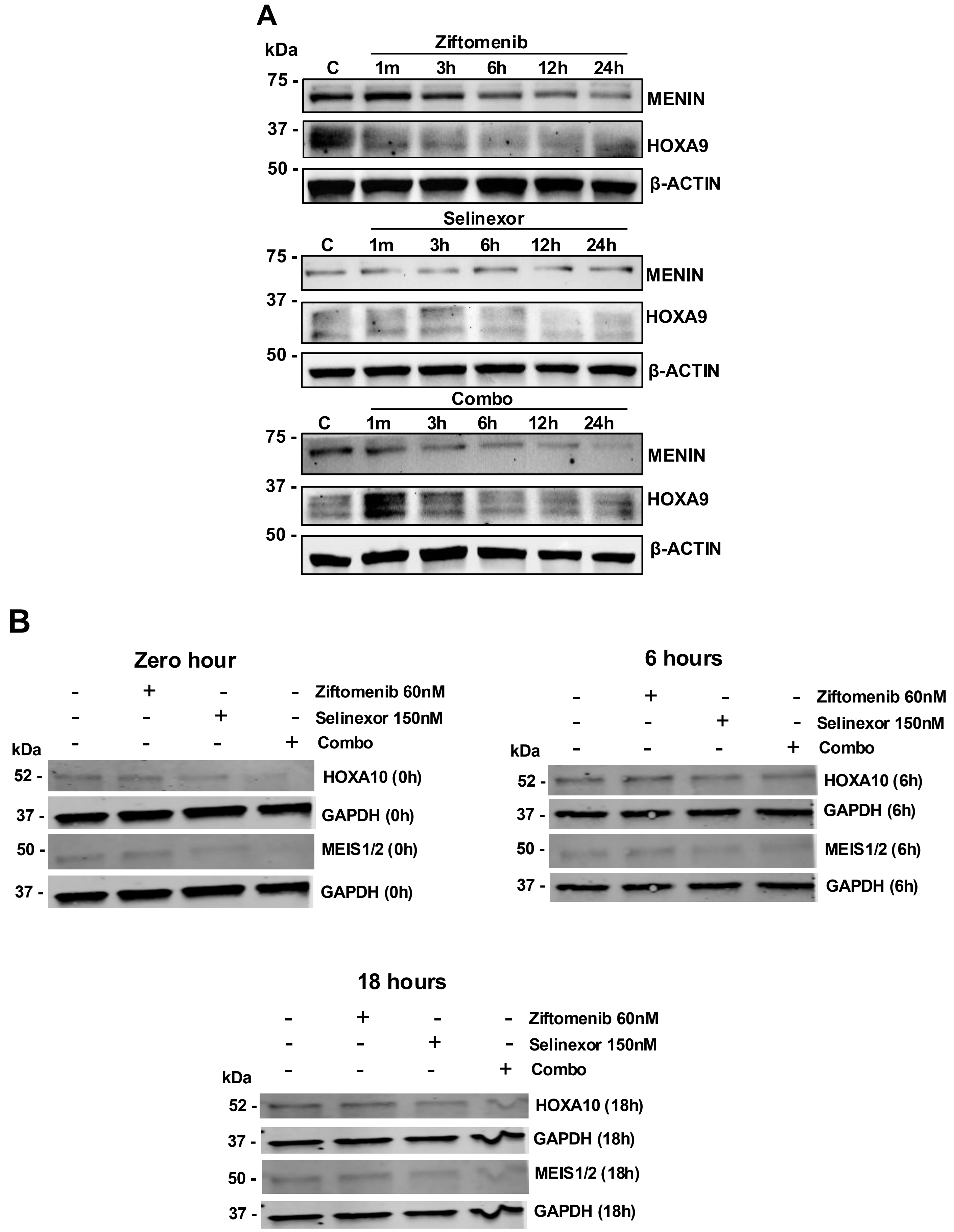
A
kDa
Ziftomenib
C
1m
3h
6h
12h
24h
75 -
MENIN
37 -
HOXA9
50 -
β-ACTIN
Selinexor
C
1m
3h
6h
12h
24h
75 -
MENIN
37 -
HOXA9
50 -
β-ACTIN
Combo
C
1m
3h
6h
12h
24h
75 -
MENIN
37 -
HOXA9
50 -
β-ACTIN
B
Zero hour
- + - - Ziftomenib 60nM
- - + - Selinexor 150nM
kDa
- - - + Combo
52 -
HOXA10 (0h)
37 -
GAPDH (0h)
50 -
MEIS1/2 (0h)
37 -
GAPDH (0h)
6 hours
- + - - Ziftomenib 60nM
- - + - Selinexor 150nM
kDa
- - - + Combo
52 -
HOXA10 (6h)
37 -
GAPDH (6h)
50 -
MEIS1/2 (6h)
37 -
GAPDH (6h)
18 hours
- + - - Ziftomenib 60nM
- - + - Selinexor 150nM
kDa
- - - + Combo
52 -
HOXA10 (18h)
37 -
GAPDH (18h)
50 -
MEIS1/2 (18h)
37 -
GAPDH (18h)

C

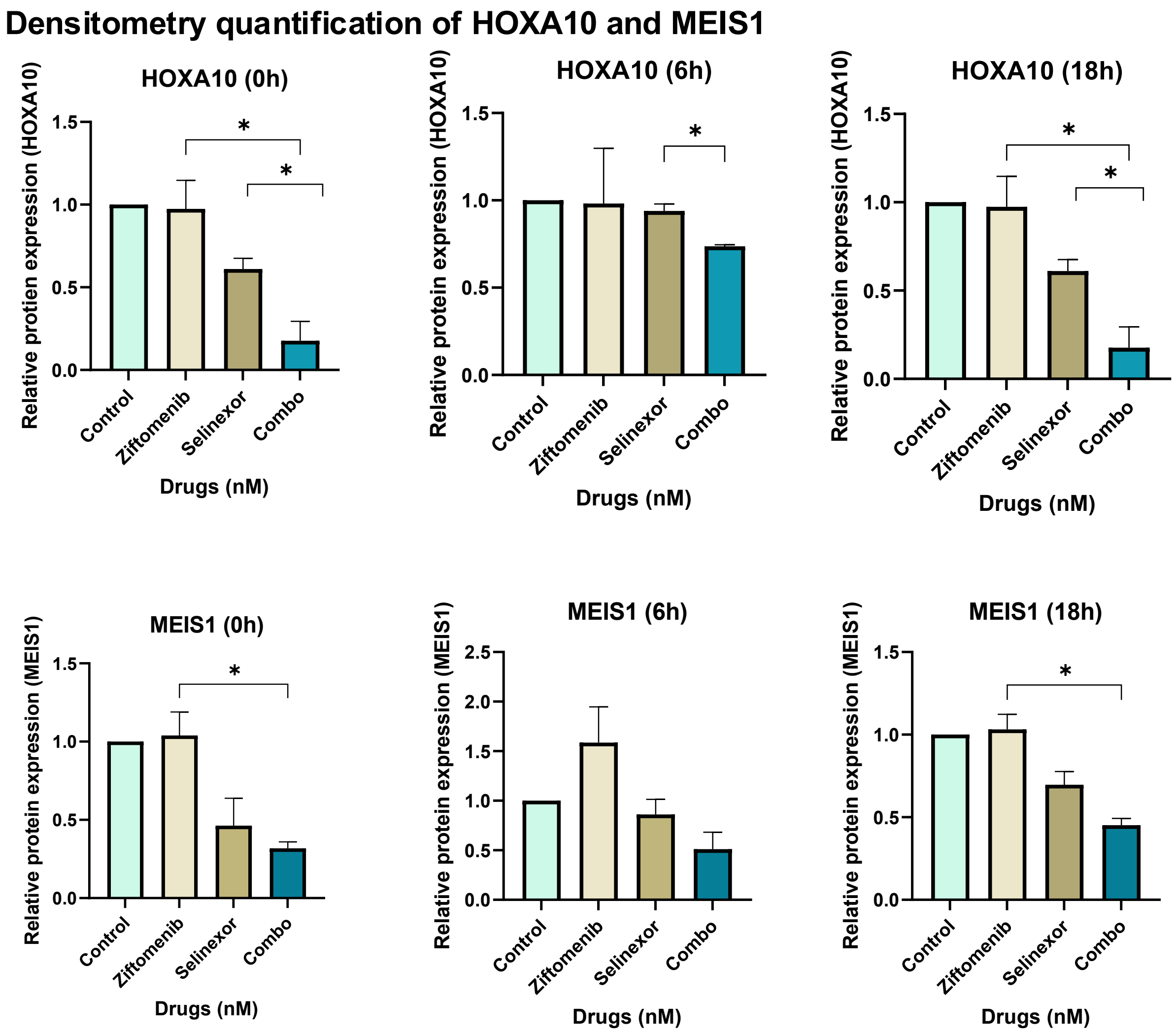

Densitometry quantification of HOXA10 and MEIS1
HOXA10 (0h)
Relative protien expression (HOXA10)
1.5
1.0
0.5
0.0
*
*
Control
Ziftomenib
Selinexor
Combo
Drugs (nM)
HOXA10 (6h)
Relative protein expression (HOXA10)
*
Drugs (nM)
HOXA10 (18h)
Relative protein expression (HOXA10)
Drugs (nM)
MEIS1 (0h)
Relative protein expression (MEIS1)
Drugs (nM)
MEIS1 (6h)
Relative protein expression (MEIS1)
2.5
2.0
Drugs (nM)
MEIS1 (18h)
Relative protein expression (MEIS1)
Drugs (nM)

Figure S6

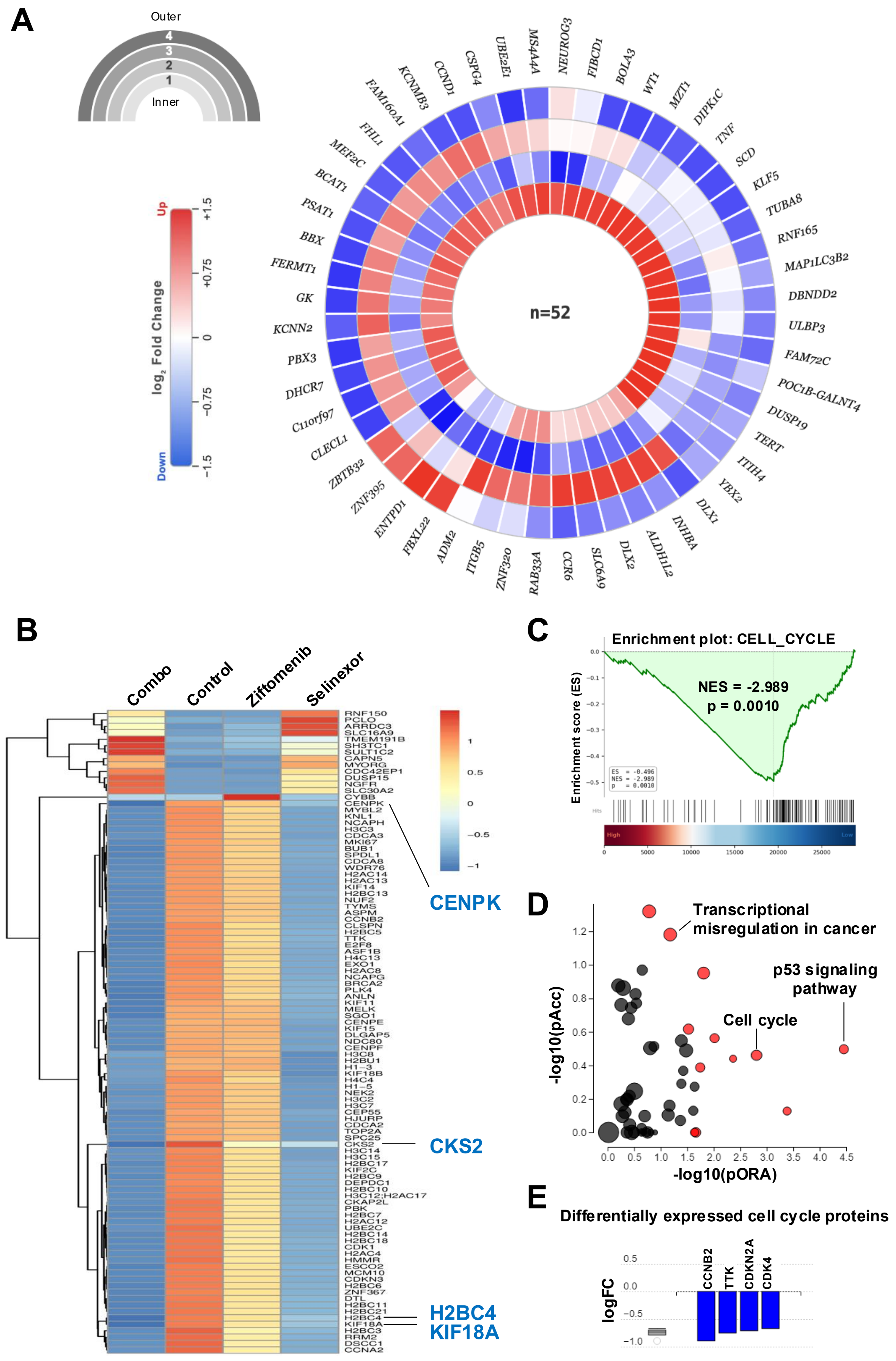
A
Outer
4
3
2
1
Inner
Up
Down
log2 Fold Change
+1.5
+0.75
0
−0.75
−1.5
n=52
B
Combo
Control
Ziftomenib
Selinexor
CENPK
CKS2
H2BC4
KIF18A
C
Enrichment plot: CELL_CYCLE
Enrichment score (ES)
NES = -2.989
p = 0.0010
D
-log10(pAcc)
-log10(pORA)
Transcriptional misregulation in cancer
p53 signaling pathway
Cell cycle
E
Differentially expressed cell cycle proteins
logFC
CCNB2
TTK
CDKN2A
CDK4

Figure S7

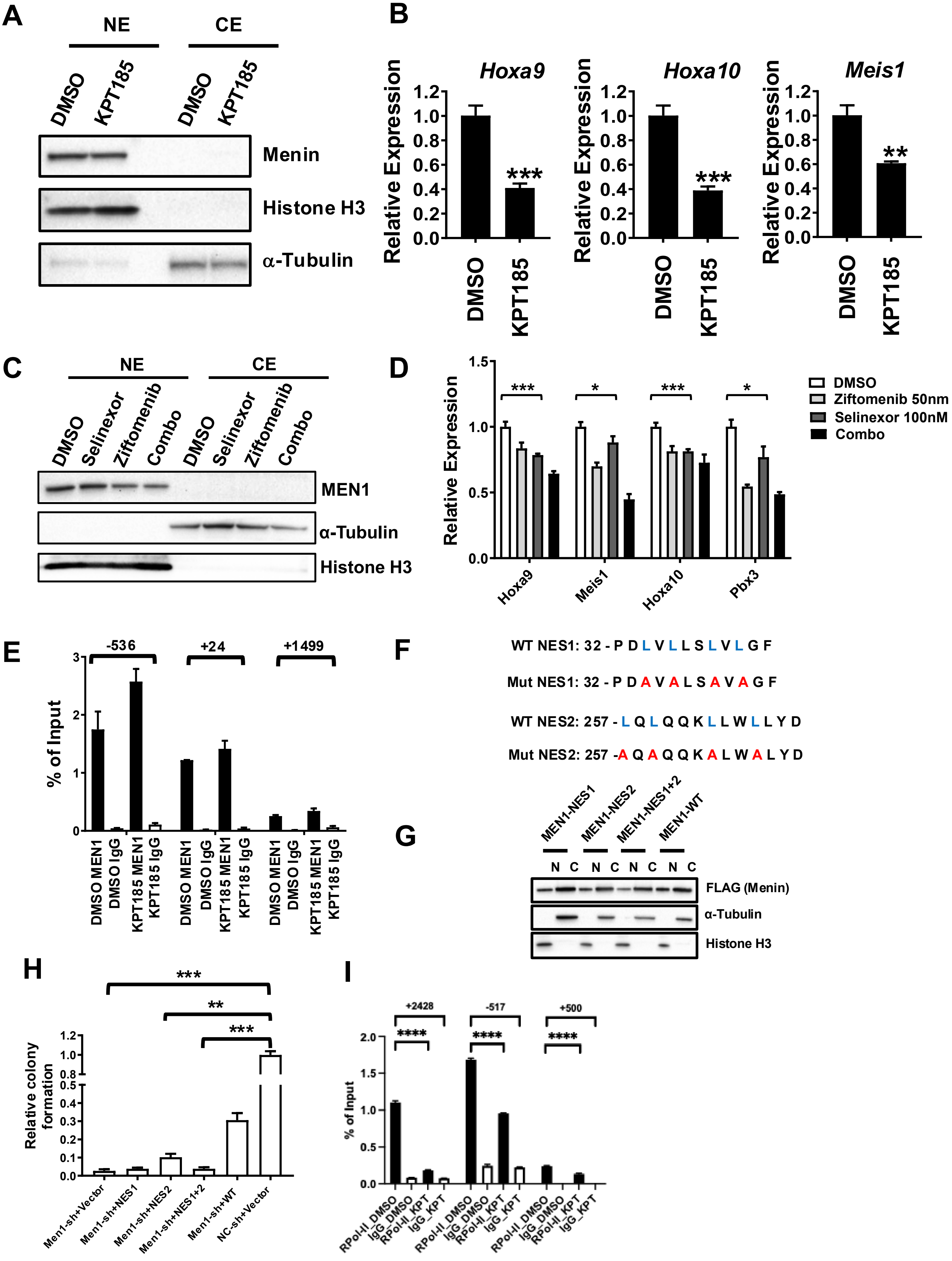
A
NE
CE
DMSO
KPT185
DMSO
KPT185
Menin
Histone H3
α-Tubulin
B
Hoxa9
Hoxa10
Meis1
Relative Expression
DMSO
KPT185
C
NE
CE
DMSO
Selinexor
Ziftomenib
Combo
MEN1
α-Tubulin
Histone H3
D
Relative Expression
DMSO
Ziftomenib 50nm
Selinexor 100nM
Combo
Hoxa9
Meis1
Hoxa10
Pbx3
E
-536
+24
+1499
% of Input
DMSO MEN1
DMSO IgG
KPT185 MEN1
KPT185 IgG
F
WT NES1: 32 - P D L V L L S L V L G F
Mut NES1: 32 - P D A V A L S A V A G F
WT NES2: 257 - L Q L Q Q K L L W L L Y D
Mut NES2: 257 -A Q A Q Q K A L W A L Y D
G
MEN1-NES1
MEN1-NES2
MEN1-NES1+2
MEN1-WT
N C N C N C N C
FLAG (Menin)
α-Tubulin
Histone H3
H
Relative colony formation
Men1-sh+Vector
Men1-sh+NES1
Men1-sh+NES2
Men1-sh+NES1+2
Men1-sh+WT
NC-sh+Vector
I
+2428
-517
+500
% of Input
RPol-II_DMSO
IgG_DMSO
RPol-II_KPT
IgG_KPT

Figure S8

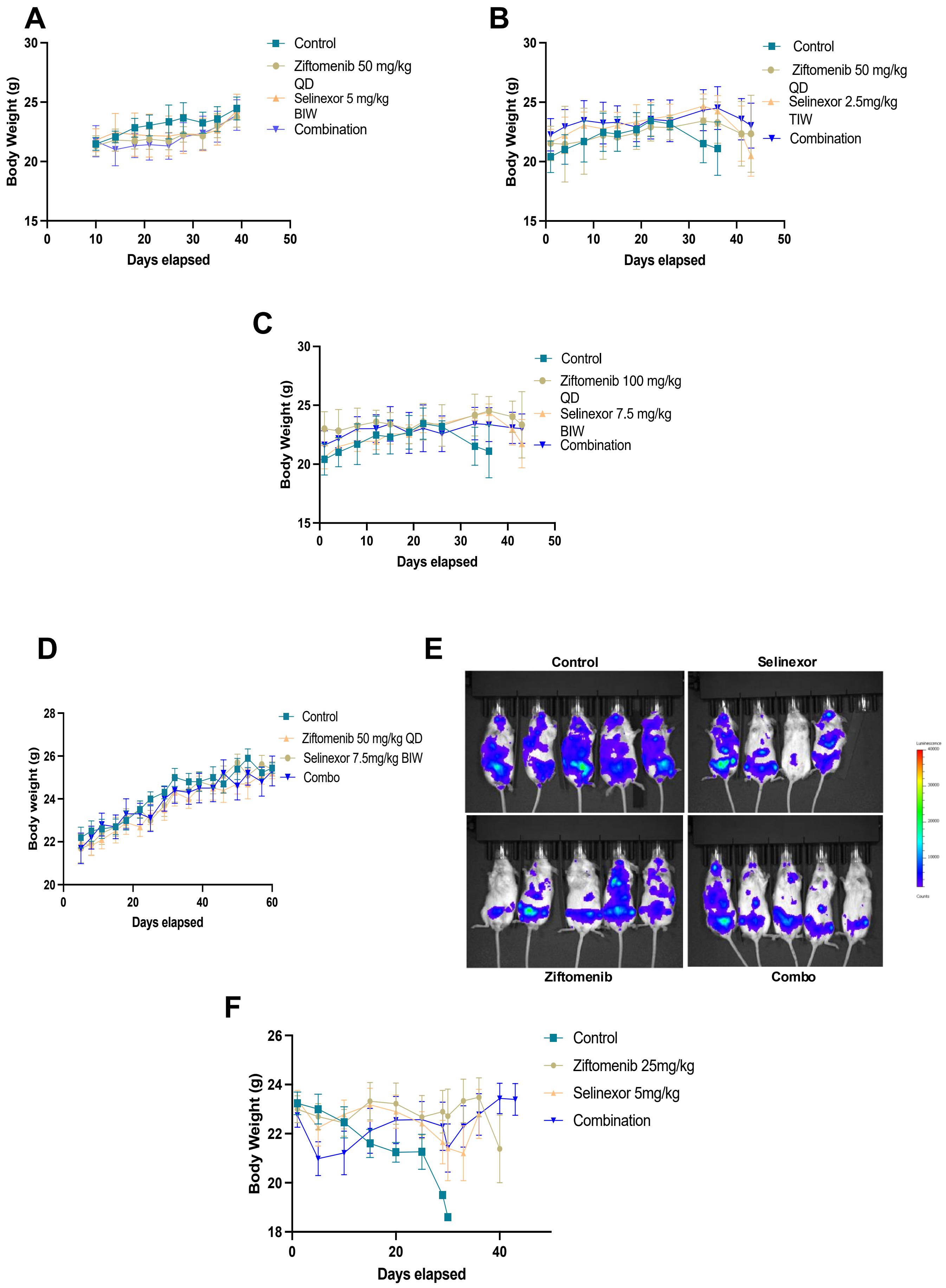
A
Body Weight (g)
Days elapsed
Control
Ziftomenib 50 mg/kg QD
Selinexor 5 mg/kg BIW
Combination
B
Body Weight (g)
Days elapsed
Control
Ziftomenib 50 mg/kg QD
Selinexor 2.5mg/kg TIW
Combination
C
Body Weight (g)
Days elapsed
Control
Ziftomenib 100 mg/kg QD
Selinexor 7.5 mg/kg BIW
Combination
D
Body weight (g)
Days elapsed
Control
Ziftomenib 50 mg/kg QD
Selinexor 7.5mg/kg BIW
Combo
E
Control
Selinexor
Ziftomenib
Combo
F
Body Weight (g)
Days elapsed
Control
Ziftomenib 25mg/kg
Selinexor 5mg/kg
Combination

Table S1. List of primers and sequences used for RT-qPCR (human).

Primers	Directions	Sequences (5'to 3')	References
GAPDH	F	CTCCTCCACCTTTGACGCTG	Shi et al., 2012
	R	ACCACCCTGTTGCTGTAGCC	Eckelhart et al., 2011
ACTIN	F	GGATGCAGAAGGAGATCACTG	Scheeren et al., 2008
	R	CGATCCACACGGAGTACTTG	
18S rRNA	F	CGGCTACCACATCCAAGGAA	Klimosch et al., 2013
	R	GCTGGAATTACCGCGGCT	
STAT5B	F	CCGGGTAAACCATGGCTGTG	Yang et al., 2024
	R	AGGAGCTGGGTGGCCTTAAT	
HOXA9	F	ATGAGAGCGGCGGAGACAAG	Faaborg et al., 2021
	R	GCACCGCTTTTTCCGAGTGG	
MEIS1	F	CAGCCCATGGGAGGTTTCGT	Bhanvadia et al., 2018
	R	GACCACCCCGGGCTACATAC	
HOXA10	F	CTTCCGAGAGCAGCAAAGCC	Song et al., 2019
	R	AGCCAGTTGGCTGCGTTTTC	
PBX3	F	ATCGGCGACATCCTCCACCAG	Cheng et al., 2020
	R	CTCATTAGCTGGGGATCGGGAG	

Table S2. List of primers and sequences used for RT-qPCR (mouse).

Primers	Directions	Sequences (5'to 3')
Hoxa9	F	CTCCTCCACCTTTGACGCTG
	R	ACCACCCTGTTGCTGTAGCC
Hoxa10	F	GGATGCAGAAGGAGATCACTG
	R	CGATCCACACGGAGTACTTG
Meis1	F	CGGCTACCACATCCAAGGAA
	R	GCTGGAATTACCGCGGCT
Pbx3	F	CCGGGTAAACCATGGCTGTG
	R	AGGAGCTGGGTGGCCTTAAT
Actb	F	ATGAGAGCGGCGGAGACAAG
	R	GCACCGCTTTTTCCGAGTGG

Table S3. List of primers and sequences used for ChIP-qPCR for *HOXA9* promoter regions (human).

Primers	Sequence
HOXA9(+2428) Forward	5’- GTGCCCACAAAGCTGTTTC-3’
HOXA9(+2428) Reverse	5’-GGGAGGAGTTGAAGGGAATG-3’
HOXA9(+517) Forward	5’-AAGTCGGAAACGACCAACAG-3’
HOXA9(+517) Reverse	5’-GCCAACCAAACACAACAGTC-3’
HOXA9(-500) Forward	5’-GCGAGGCAAACGAATCTGTT-3’
HOXA9(-500) Reverse	5’-CCAAATCGCATTGTCGCTCT-3’

Table S4. List of primers and sequences used for ChIP-qPCR for *HOXA9* promoter regions (mouse).

Primers	Sequence
Hoxa9(-536) Forward	5′- TGTCAGAGCGTTGGAAAGTG-3′
Hoxa9(-536) Reverse	5′- TGTGAATTTTGTGCCTTCCA-3′
Hoxa9(+24) Forward	5′- ACCAGAGCGGTTCATACAGG-3′
Hoxa9(+24) Reverse	5′- CAGACTGGAGATGGGGAAAA-3′
Hoxa9(+1499) Forward	5′- TGCCTGCTGCAGTGTATCAT-3′
Hoxa9(+1499) Reverse	5′- GAGCGGTTCAGGTTTAATGC-3′
Hoxa9(+1612) Forward	5’- GGTGCGCTCTCCTTCGC-3’
Hoxa9(+1612) Reverse	5’- GCATAGTCAGTCAGGGACAAAGTG-3’

Table S5. Combination Index (CI) of *KMT2A*-r primary leukemic samples

Primary Patient Samples	Ziftomenib (nM)	Selinexor (nM)	Effect	CI
KCI-I	1000<br>500<br>250	100<br>50<br>25	0.48267<br>0.36344<br>0.29209	0.68414<br>0.78183<br>0.70335
KCI-II	1000<br>500<br>250	150<br>75<br>37	0.57320<br>0.59531<br>0.51281	0.49262<br>0.21725<br>0.17410
KCI-III	1000<br>125<br>62.5	100<br>12.5<br>6.25	0.65075<br>0.36888<br>0.30623	0.58622<br>0.73140<br>0.63841
KCI-IV	1000<br>500<br>125	150<br>75<br>18.75	0.87332<br>0.82221<br>0.75300	0.15495<br>0.15146<br>0.12842
KCI-V	1000<br>500<br>250	150<br>75<br>37	0.59772<br>0.48959<br>0.32524	0.53675<br>0.47235<br>0.58009
KCI-VI	1000<br>500.0<br>250	150<br>75<br>37.5	0.76597<br>0.70604<br>0.56949	0.50069<br>0.42929<br>0.60721
KCI-VII	1000<br>500<br>250	150<br>75<br>37.5	0.63951<br>0.48227<br>0.33596	0.74455<br>0.64496<br>0.54580
KCI-VIII	1000<br>500	150<br>75	0.32810<br>0.19539	1.18316<br>0.83296
KCI-IX	1000<br>500<br>250	150<br>75<br>37.5	0.79545<br>0.70125<br>0.61975	0.13173<br>0.24258<br>0.31200

Table S6. Combination Index (CI) of *NPM1*-m primary leukemic samples

Primary Patient Samples	Ziftomenib (nM)	Selinexor (nM)	Effect	CI
NPM-I	2000<br>1000<br>500	200<br>100<br>50	0.66787<br>0.51051<br>0.35039	0.28434<br>0.49038<br>0.85192
NPM-II	2000<br>250<br>62.5	200<br>25<br>6.25	0.82901<br>0.34504<br>0.24198	0.64878<br>0.95634<br>0.43218
NPM-III	2000<br>1000<br>500	200<br>100<br>50	0.80567<br>0.73987<br>0.59586	0.38499<br>0.30577<br>0.40935
NPM-IV	2000<br>1000<br>500	200<br>100<br>50	0.65242<br>0.49634<br>0.39191	0.27374<br>0.71277<br>4.78592
NPM-V	2000<br>1000<br>500	100<br>50<br>25	0.56037<br>0.41558<br>0.41727	0.93001<br>1.17843<br>0.58230

Table S7. Cytogenetics, translocations and mutations of the *KMT2A*-r primary leukemic samples

KMT2A-r sample	Cytogenetics at sample time point	Translocations	Associated Mutations
KCI-I	46,XX,t(1;9;11)(q12;p22;q23)/47, idem,+11	MLL(KMT2A)/11q23 in 86.5% of cells	None
KCI-II	47+R+48, XY, der(1)t(1;1)(p13;q25)t(1;9)(q21;q13),-6,+8, t(11;19)(q23;p13.3), +r, +mar[cp19]/46,XY	MLL(KMT2A)/11q23 in 75% of cells	None
KCI-III	46, XY, t(6;11)(q27;q23)/46,XY	MLL/11q23 gene in 87% of cells	ETV6, BCOR
KCI-IV	47,XX,+X,t(4;11)(q21;q23),(p13)/46,XX	"MLL(KMT2A)/11q23 gene region in 93% of cells"	None
KCI-V	46 XX, t(9,11)(p22;q23)/46, XX	MLL/11q23 36% of cell	None
KCI-VI	46 XX, t(9,11)(p22;q23)[12]/46, XX	MLL/11q23 gene in 88.5% cells	FLT3-ITD, STAG2
KCI-VII	48 XX, -7, +8, +8, +8, t(11,17)(q23;q25)	MLL/11q23 92% of cell	NRAS
KCI-VIII	46 XY	MLL/11q23 87% of cell	IDH2, IDH2
KCI-IX	46,XY,t(9,11)(p22;q23)	MLL/11q23 86% of cell	None

Table S8. Cytogenetics, translocations and mutations of the *NPM1*-m primary leukemic samples

NPM-m sample	Cytogenetics at sample time point	Translocations	Associated Mutations
NPM-I	46,XX,t(1;9;11)(q12;p22;q23)/47, idem,+11	MLL(KMT2A)/11q23 in 86.5% of cells	None
NPM-II	47+R+48, XY, der(1)t(1;1)(p13;q25)t(1;9)(q21;q13),-6,+8, t(11;19)(q23;p13.3), +r, +mar[cp19]/46,XY	MLL(KMT2A)/11q23 in 75% of cells	None
NPM-III	46, XY, t(6;11)(q27;q23)/46,XY	MLL/11q23 gene in 87% of cells	ETV6, BCOR
NPM-IV	47,XX,+X,t(4;11)(q21;q23),(p13)/46,XX	"MLL(KMT2A)/11q23 gene region in 93% of cells"	None
NPM-V	46 XX, t(9,11)(p22;q23)/46, XX	MLL/11q23 36% of cell	None
